# Supplementary material for: ‘Candidatus Phytoplasma zeae’: community-driven species delineation of the maize bushy stunt phytoplasma, a Dalbulus-transmitted corn pathogen confined to the Americas
Source: Int J Syst Evol Microbiol. 2026 Oct 9;76(10):007301. doi: 10.1099/ijsem.0.007301 (PMC13660524; doi:10.1099/ijsem.0.007301)
Supplement: Supplementary file 1 [file ijsem-76-07301-s001.pdf]

Table S1. Occurrences of *Dalbulus maidis, D. elimatus,* and Maize bushy stunt phytoplasma (MBSP). When a reference only provided the locality name (without coordinates), the record was registered based on this locality, not the exact point. For the MBSP, only references that included the molecular identification level were included. Those based solely on symptomatic plants were excluded due to similarities with corn stunt spiroplasma symptoms.

Species	Latitude	Longitude	Country	Reference
D. maidis	-32.60432	-64.38400	Argentina	Virla et al. 2010; Virla et al. 2013
D. maidis	-31.06889	-64.12670	Argentina	Virla et al. 2010; Virla et al. 2013
D. maidis	-30.92053	-64.07542	Argentina	Virla et al. 2010; Virla et al. 2013
D. maidis	-29.89343	-63.72887	Argentina	Virla et al. 2010; Virla et al. 2013
D. maidis	-27.07337	-61.07945	Argentina	Virla et al. 2010; Virla et al. 2013
D. maidis	-26.83430	-65.27381	Argentina	Virla et al. 2010; Virla et al. 2013
D. maidis	-26.60750	-64.80910	Argentina	Santana Jr et al. 2019
D. maidis	-26.65040	-64.54690	Argentina	Santana Jr et al. 2019
D. maidis	-26.55070	-64.64080	Argentina	Santana Jr et al. 2019
D. maidis	-26.45410	-64.56370	Argentina	Santana Jr et al. 2019
D. maidis	-26.35090	-64.63030	Argentina	Santana Jr et al. 2019
D. maidis	-26.24900	-64.52470	Argentina	Santana Jr et al. 2019
D. maidis	-26.02220	-64.22620	Argentina	Santana Jr et al. 2019
D. maidis	-25.96320	-63.95830	Argentina	Santana Jr et al. 2019
D. maidis	-26.17850	-63.85010	Argentina	Santana Jr et al. 2019
D. maidis	-25.64420	-64.08810	Argentina	Santana Jr et al. 2019
D. maidis	-25.75620	-64.55930	Argentina	Santana Jr et al. 2019
D. maidis	-25.78530	-64.81340	Argentina	Santana Jr et al. 2019
D. maidis	-25.61170	-64.67000	Argentina	Santana Jr et al. 2019
D. maidis	-25.42550	-64.81180	Argentina	Santana Jr et al. 2019
D. maidis	-25.13230	-64.22660	Argentina	Santana Jr et al. 2019
D. maidis	-25.18250	-63.78050	Argentina	Santana Jr et al. 2019
D. maidis	-25.20220	-63.26650	Argentina	Santana Jr et al. 2019
D. maidis	-24.77810	-62.84880	Argentina	Santana Jr et al. 2019
D. maidis	-24.41500	-64.01950	Argentina	Santana Jr et al. 2019
D. maidis	-24.02410	-63.92300	Argentina	Santana Jr et al. 2019
D. maidis	-23.62030	-64.09760	Argentina	Santana Jr et al. 2019

D. maidis	-23.14390	-63.84420	Argentina	Santana Jr et al. 2019
D. maidis	-22.60420	-63.44600	Argentina	Santana Jr et al. 2019
D. maidis	-24.53050	-62.08960	Argentina	Santana Jr et al. 2019
D. maidis	-24.87270	-61.54070	Argentina	Santana Jr et al. 2019
D. maidis	-25.42120	-61.86010	Argentina	Santana Jr et al. 2019
D. maidis	-25.78730	-61.55420	Argentina	Santana Jr et al. 2019
D. maidis	-26.13370	-60.97560	Argentina	Santana Jr et al. 2019
D. maidis	-26.61870	-61.31730	Argentina	Santana Jr et al. 2019
D. maidis	-18.22506	-63.84817	Bolivia	Zahniser 2007
D. maidis	-17.41839	-66.20111	Bolivia	Zahniser 2007
D. maidis	-17.41667	-63.25000	Bolivia	Zahniser 2007
D. maidis	-17.36347	-66.32128	Bolivia	Zahniser 2007
D. maidis	-28.49078	-52.55178	Brazil	de Oliveira et al. 2007; Meneses et al. 2016; Oliveira et al. 2013a; Oliveira et al. 2013b; Waquil et al. 1999
D. maidis	-28.13898	-52.29003	Brazil	de Oliveira et al. 2007; Meneses et al. 2016; Oliveira et al. 2013a; Oliveira et al. 2013b; Waquil et al. 1999
D. maidis	-27.64573	-53.32819	Brazil	de Oliveira et al. 2007; Meneses et al. 2016; Oliveira et al. 2013a; Oliveira et al. 2013b; Waquil et al. 1999
D. maidis	-27.37891	-53.40049	Brazil	de Oliveira et al. 2007; Meneses et al. 2016; Oliveira et al. 2013a; Oliveira et al. 2013b; Waquil et al. 1999
D. maidis	-27.07296	-52.70312	Brazil	de Oliveira et al. 2007; Meneses et al. 2016; Oliveira et al. 2013a; Oliveira et al. 2013b; Waquil et al. 1999
D. maidis	-27.04991	-52.61833	Brazil	de Oliveira et al. 2007; Meneses et al. 2016; Oliveira et al. 2013a; Oliveira et al. 2013b; Waquil et al. 1999
D. maidis	-26.99751	-51.87348	Brazil	de Oliveira et al. 2007; Meneses et al. 2016; Oliveira et al. 2013a; Oliveira et al. 2013b; Waquil et al. 1999
D. maidis	-26.89422	-52.92124	Brazil	de Oliveira et al. 2007; Meneses et al. 2016; Oliveira et al. 2013a; Oliveira et al. 2013b; Waquil et al. 1999
D. maidis	-26.87883	-53.17009	Brazil	de Oliveira et al. 2007; Meneses et al. 2016; Oliveira et al. 2013a; Oliveira et al. 2013b; Waquil et al. 1999
D. maidis	-26.82675	-53.26586	Brazil	de Oliveira et al. 2007; Meneses et al. 2016; Oliveira et al. 2013a; Oliveira et al. 2013b; Waquil et al. 1999
D. maidis	-26.69481	-53.51712	Brazil	de Oliveira et al. 2007; Meneses et al. 2016; Oliveira et al. 2013a; Oliveira et al. 2013b; Waquil et al. 1999
D. maidis	-26.58171	-53.52164	Brazil	de Oliveira et al. 2007; Meneses et al. 2016; Oliveira et al. 2013a; Oliveira et al. 2013b; Waquil et al. 1999
D. maidis	-26.46770	-53.50355	Brazil	de Oliveira et al. 2007; Meneses et al. 2016; Oliveira et al. 2013a; Oliveira et al. 2013b; Waquil et al. 1999
D. maidis	-26.39129	-53.51970	Brazil	de Oliveira et al. 2007; Meneses et al. 2016; Oliveira et al. 2013a; Oliveira et al. 2013b; Waquil et al. 1999
D. maidis	-26.20907	-53.63643	Brazil	de Oliveira et al. 2007; Meneses et al. 2016; Oliveira et al. 2013a; Oliveira et al. 2013b; Waquil et al. 1999
D. maidis	-26.07725	-53.71153	Brazil	de Oliveira et al. 2007; Meneses et al. 2016; Oliveira et al. 2013a; Oliveira et al. 2013b; Waquil et al. 1999
D. maidis	-25.90569	-50.44474	Brazil	de Oliveira et al. 2007; Meneses et al. 2016; Oliveira et al. 2013a; Oliveira et al. 2013b; Waquil et al. 1999
D. maidis	-25.75797	-49.74333	Brazil	de Oliveira et al. 2007; Meneses et al. 2016; Oliveira et al. 2013a; Oliveira et al. 2013b; Waquil et al. 1999
D. maidis	-25.69775	-53.77579	Brazil	de Oliveira et al. 2007; Meneses et al. 2016; Oliveira et al. 2013a; Oliveira et al. 2013b; Waquil et al. 1999
D. maidis	-25.02648	-53.57596	Brazil	de Oliveira et al. 2007; Meneses et al. 2016; Oliveira et al. 2013a; Oliveira et al. 2013b; Waquil et al. 1999

D. maidis	-24.65008	-53.74867	Brazil	de Oliveira et al. 2007; Meneses et al. 2016; Oliveira et al. 2013a; Oliveira et al. 2013b; Waquil et al. 1999
D. maidis	-24.64628	-53.29883	Brazil	de Oliveira et al. 2007; Meneses et al. 2016; Oliveira et al. 2013a; Oliveira et al. 2013b; Waquil et al. 1999
D. maidis	-24.41319	-52.81781	Brazil	de Oliveira et al. 2007; Meneses et al. 2016; Oliveira et al. 2013a; Oliveira et al. 2013b; Waquil et al. 1999
D. maidis	-23.99145	-52.35483	Brazil	de Oliveira et al. 2007; Meneses et al. 2016; Oliveira et al. 2013a; Oliveira et al. 2013b; Waquil et al. 1999
D. maidis	-23.56396	-51.53198	Brazil	de Oliveira et al. 2007; Meneses et al. 2016; Oliveira et al. 2013a; Oliveira et al. 2013b; Waquil et al. 1999
D. maidis	-23.51305	-51.71274	Brazil	de Oliveira et al. 2007; Meneses et al. 2016; Oliveira et al. 2013a; Oliveira et al. 2013b; Waquil et al. 1999
D. maidis	-23.35021	-51.89945	Brazil	de Oliveira et al. 2007; Meneses et al. 2016; Oliveira et al. 2013a; Oliveira et al. 2013b; Waquil et al. 1999
D. maidis	-23.25878	-51.26167	Brazil	de Oliveira et al. 2007; Meneses et al. 2016; Oliveira et al. 2013a; Oliveira et al. 2013b; Waquil et al. 1999
D. maidis	-23.16638	-50.61167	Brazil	de Oliveira et al. 2007; Meneses et al. 2016; Oliveira et al. 2013a; Oliveira et al. 2013b; Waquil et al. 1999
D. maidis	-23.14984	-50.53593	Brazil	de Oliveira et al. 2007; Meneses et al. 2016; Oliveira et al. 2013a; Oliveira et al. 2013b; Waquil et al. 1999
D. maidis	-22.80343	-47.62894	Brazil	de Oliveira et al. 2007; Meneses et al. 2016; Oliveira et al. 2013a; Oliveira et al. 2013b; Waquil et al. 1999
D. maidis	-22.25437	-45.88173	Brazil	de Oliveira et al. 2007; Meneses et al. 2016; Oliveira et al. 2013a; Oliveira et al. 2013b; Waquil et al. 1999
D. maidis	-22.13533	-45.74988	Brazil	de Oliveira et al. 2007; Meneses et al. 2016; Oliveira et al. 2013a; Oliveira et al. 2013b; Waquil et al. 1999
D. maidis	-21.90188	-45.62874	Brazil	de Oliveira et al. 2007; Meneses et al. 2016; Oliveira et al. 2013a; Oliveira et al. 2013b; Waquil et al. 1999
D. maidis	-21.65465	-45.31596	Brazil	de Oliveira et al. 2007; Meneses et al. 2016; Oliveira et al. 2013a; Oliveira et al. 2013b; Waquil et al. 1999
D. maidis	-21.40371	-44.92868	Brazil	de Oliveira et al. 2007; Meneses et al. 2016; Oliveira et al. 2013a; Oliveira et al. 2013b; Waquil et al. 1999
D. maidis	-21.21078	-45.05888	Brazil	de Oliveira et al. 2007; Meneses et al. 2016; Oliveira et al. 2013a; Oliveira et al. 2013b; Waquil et al. 1999
D. maidis	-21.01423	-47.33700	Brazil	de Oliveira et al. 2007; Meneses et al. 2016; Oliveira et al. 2013a; Oliveira et al. 2013b; Waquil et al. 1999
D. maidis	-20.87736	-46.37156	Brazil	de Oliveira et al. 2007; Meneses et al. 2016; Oliveira et al. 2013a; Oliveira et al. 2013b; Waquil et al. 1999
D. maidis	-20.50531	-55.83438	Brazil	de Oliveira et al. 2007; Meneses et al. 2016; Oliveira et al. 2013a; Oliveira et al. 2013b; Waquil et al. 1999
D. maidis	-20.46559	-45.80249	Brazil	de Oliveira et al. 2007; Meneses et al. 2016; Oliveira et al. 2013a; Oliveira et al. 2013b; Waquil et al. 1999
D. maidis	-19.86448	-47.47283	Brazil	de Oliveira et al. 2007; Meneses et al. 2016; Oliveira et al. 2013a; Oliveira et al. 2013b; Waquil et al. 1999
D. maidis	-19.79104	-45.63820	Brazil	de Oliveira et al. 2007; Meneses et al. 2016; Oliveira et al. 2013a; Oliveira et al. 2013b; Waquil et al. 1999
D. maidis	-19.57538	-47.00335	Brazil	de Oliveira et al. 2007; Meneses et al. 2016; Oliveira et al. 2013a; Oliveira et al. 2013b; Waquil et al. 1999
D. maidis	-19.45972	-44.17529	Brazil	de Oliveira et al. 2007; Meneses et al. 2016; Oliveira et al. 2013a; Oliveira et al. 2013b; Waquil et al. 1999
D. maidis	-19.18809	-48.15771	Brazil	de Oliveira et al. 2007; Meneses et al. 2016; Oliveira et al. 2013a; Oliveira et al. 2013b; Waquil et al. 1999
D. maidis	-19.17739	-47.68901	Brazil	de Oliveira et al. 2007; Meneses et al. 2016; Oliveira et al. 2013a; Oliveira et al. 2013b; Waquil et al. 1999
D. maidis	-18.11894	-49.24617	Brazil	de Oliveira et al. 2007; Meneses et al. 2016; Oliveira et al. 2013a; Oliveira et al. 2013b; Waquil et al. 1999
D. maidis	-15.69038	-47.78185	Brazil	de Oliveira et al. 2007; Meneses et al. 2016; Oliveira et al. 2013a; Oliveira et al. 2013b; Waquil et al. 1999
D. maidis	-8.49614	-45.77746	Brazil	de Oliveira et al. 2007; Meneses et al. 2016; Oliveira et al. 2013a; Oliveira et al. 2013b; Waquil et al. 1999
D. maidis	-7.64006	-46.34842	Brazil	de Oliveira et al. 2007; Meneses et al. 2016; Oliveira et al. 2013a; Oliveira et al. 2013b; Waquil et al. 1999
D. maidis	-7.57294	-45.96202	Brazil	de Oliveira et al. 2007; Meneses et al. 2016; Oliveira et al. 2013a; Oliveira et al. 2013b; Waquil et al. 1999

D. maidis	-6.98939	-45.43294	Brazil	de Oliveira et al. 2007; Meneses et al. 2016; Oliveira et al. 2013a; Oliveira et al. 2013b; Waquil et al. 1999
D. maidis	-5.23151	-37.38243	Brazil	de Oliveira et al. 2007; Meneses et al. 2016; Oliveira et al. 2013a; Oliveira et al. 2013b; Waquil et al. 1999
D. maidis	-5.03159	-42.79951	Brazil	de Oliveira et al. 2007; Meneses et al. 2016; Oliveira et al. 2013a; Oliveira et al. 2013b; Waquil et al. 1999
D. maidis	-17.87615	-51.66616	Brazil	de Oliveira et al. 2007; Meneses et al. 2016; Oliveira et al. 2013a; Oliveira et al. 2013b; Waquil et al. 1999
D. maidis	-13.05170	-55.87118	Brazil	de Oliveira et al. 2007; Meneses et al. 2016; Oliveira et al. 2013a; Oliveira et al. 2013b; Waquil et al. 1999
D. maidis	-16.80813	-47.61953	Brazil	de Oliveira et al. 2007; Meneses et al. 2016; Oliveira et al. 2013a; Oliveira et al. 2013b; Waquil et al. 1999
D. maidis	-12.53862	-55.67438	Brazil	de Oliveira et al. 2007; Meneses et al. 2016; Oliveira et al. 2013a; Oliveira et al. 2013b; Waquil et al. 1999
D. maidis	-19.78650	-47.88372	Brazil	de Oliveira et al. 2007; Meneses et al. 2016; Oliveira et al. 2013a; Oliveira et al. 2013b; Waquil et al. 1999
D. maidis	-18.38343	-52.66614	Brazil	de Oliveira et al. 2007; Meneses et al. 2016; Oliveira et al. 2013a; Oliveira et al. 2013b; Waquil et al. 1999
D. maidis	-17.80668	-50.88779	Brazil	de Oliveira et al. 2007; Meneses et al. 2016; Oliveira et al. 2013a; Oliveira et al. 2013b; Waquil et al. 1999
D. maidis	-16.33276	-46.91046	Brazil	de Oliveira et al. 2007; Meneses et al. 2016; Oliveira et al. 2013a; Oliveira et al. 2013b; Waquil et al. 1999
D. maidis	-12.39517	-45.21213	Brazil	de Oliveira et al. 2007; Meneses et al. 2016; Oliveira et al. 2013a; Oliveira et al. 2013b; Waquil et al. 1999
D. maidis	-24.78629	-49.77244	Brazil	de Oliveira et al. 2007; Meneses et al. 2016; Oliveira et al. 2013a; Oliveira et al. 2013b; Waquil et al. 1999
D. maidis	-25.41567	-51.52491	Brazil	de Oliveira et al. 2007; Meneses et al. 2016; Oliveira et al. 2013a; Oliveira et al. 2013b; Waquil et al. 1999
D. maidis	-25.48727	-50.62241	Brazil	de Oliveira et al. 2007; Meneses et al. 2016; Oliveira et al. 2013a; Oliveira et al. 2013b; Waquil et al. 1999
D. maidis	-23.98934	-48.84611	Brazil	de Oliveira et al. 2007; Meneses et al. 2016; Oliveira et al. 2013a; Oliveira et al. 2013b; Waquil et al. 1999
D. maidis	-24.50791	-50.39158	Brazil	de Oliveira et al. 2007; Meneses et al. 2016; Oliveira et al. 2013a; Oliveira et al. 2013b; Waquil et al. 1999
D. maidis	-17.29163	-51.23430	Brazil	de Oliveira et al. 2007; Meneses et al. 2016; Oliveira et al. 2013a; Oliveira et al. 2013b; Waquil et al. 1999
D. maidis	-27.39065	-51.24018	Brazil	de Oliveira et al. 2007; Meneses et al. 2016; Oliveira et al. 2013a; Oliveira et al. 2013b; Waquil et al. 1999
D. maidis	-24.88558	-53.48617	Brazil	de Oliveira et al. 2007; Meneses et al. 2016; Oliveira et al. 2013a; Oliveira et al. 2013b; Waquil et al. 1999
D. maidis	-24.76918	-51.73829	Brazil	de Oliveira et al. 2007; Meneses et al. 2016; Oliveira et al. 2013a; Oliveira et al. 2013b; Waquil et al. 1999
D. maidis	-12.58292	-56.23058	Brazil	de Oliveira et al. 2007; Meneses et al. 2016; Oliveira et al. 2013a; Oliveira et al. 2013b; Waquil et al. 1999
D. maidis	-15.31531	-54.78717	Brazil	de Oliveira et al. 2007; Meneses et al. 2016; Oliveira et al. 2013a; Oliveira et al. 2013b; Waquil et al. 1999
D. maidis	-23.83644	-49.15704	Brazil	de Oliveira et al. 2007; Meneses et al. 2016; Oliveira et al. 2013a; Oliveira et al. 2013b; Waquil et al. 1999
D. maidis	-15.98570	-47.56006	Brazil	de Oliveira et al. 2007; Meneses et al. 2016; Oliveira et al. 2013a; Oliveira et al. 2013b; Waquil et al. 1999
D. maidis	-13.56164	-58.89434	Brazil	de Oliveira et al. 2007; Meneses et al. 2016; Oliveira et al. 2013a; Oliveira et al. 2013b; Waquil et al. 1999
D. maidis	-15.48625	-54.29713	Brazil	de Oliveira et al. 2007; Meneses et al. 2016; Oliveira et al. 2013a; Oliveira et al. 2013b; Waquil et al. 1999
D. maidis	-22.27715	-54.77588	Brazil	de Oliveira et al. 2007; Meneses et al. 2016; Oliveira et al. 2013a; Oliveira et al. 2013b; Waquil et al. 1999
D. maidis	-19.38624	-47.35386	Brazil	de Oliveira et al. 2007; Meneses et al. 2016; Oliveira et al. 2013a; Oliveira et al. 2013b; Waquil et al. 1999
D. maidis	-25.12491	-50.23779	Brazil	de Oliveira et al. 2007; Meneses et al. 2016; Oliveira et al. 2013a; Oliveira et al. 2013b; Waquil et al. 1999
D. maidis	-25.48198	-51.90455	Brazil	de Oliveira et al. 2007; Meneses et al. 2016; Oliveira et al. 2013a; Oliveira et al. 2013b; Waquil et al. 1999
D. maidis	-18.73271	-52.63871	Brazil	de Oliveira et al. 2007; Meneses et al. 2016; Oliveira et al. 2013a; Oliveira et al. 2013b; Waquil et al. 1999

D. maidis	-21.59274	-55.16701	Brazil	de Oliveira et al. 2007; Meneses et al. 2016; Oliveira et al. 2013a; Oliveira et al. 2013b; Waquil et al. 1999
D. maidis	-16.29586	-47.87334	Brazil	de Oliveira et al. 2007; Meneses et al. 2016; Oliveira et al. 2013a; Oliveira et al. 2013b; Waquil et al. 1999
D. maidis	-12.16623	-45.72854	Brazil	de Oliveira et al. 2007; Meneses et al. 2016; Oliveira et al. 2013a; Oliveira et al. 2013b; Waquil et al. 1999
D. maidis	-17.50559	-52.55606	Brazil	de Oliveira et al. 2007; Meneses et al. 2016; Oliveira et al. 2013a; Oliveira et al. 2013b; Waquil et al. 1999
D. maidis	-19.20680	-54.59664	Brazil	de Oliveira et al. 2007; Meneses et al. 2016; Oliveira et al. 2013a; Oliveira et al. 2013b; Waquil et al. 1999
D. maidis	-17.51884	-52.22939	Brazil	de Oliveira et al. 2007; Meneses et al. 2016; Oliveira et al. 2013a; Oliveira et al. 2013b; Waquil et al. 1999
D. maidis	-25.73546	-51.70264	Brazil	de Oliveira et al. 2007; Meneses et al. 2016; Oliveira et al. 2013a; Oliveira et al. 2013b; Waquil et al. 1999
D. maidis	-22.62469	-54.80386	Brazil	de Oliveira et al. 2007; Meneses et al. 2016; Oliveira et al. 2013a; Oliveira et al. 2013b; Waquil et al. 1999
D. maidis	-15.64023	-46.45817	Brazil	de Oliveira et al. 2007; Meneses et al. 2016; Oliveira et al. 2013a; Oliveira et al. 2013b; Waquil et al. 1999
D. maidis	-18.54108	-52.94955	Brazil	de Oliveira et al. 2007; Meneses et al. 2016; Oliveira et al. 2013a; Oliveira et al. 2013b; Waquil et al. 1999
D. maidis	-31.38726	-52.71554	Brazil	de Oliveira et al. 2007; Meneses et al. 2016; Oliveira et al. 2013a; Oliveira et al. 2013b; Waquil et al. 1999
D. maidis	-26.15373	-49.78481	Brazil	de Oliveira et al. 2007; Meneses et al. 2016; Oliveira et al. 2013a; Oliveira et al. 2013b; Waquil et al. 1999
D. maidis	-24.96368	-50.12373	Brazil	de Oliveira et al. 2007; Meneses et al. 2016; Oliveira et al. 2013a; Oliveira et al. 2013b; Waquil et al. 1999
D. maidis	-25.35752	-54.26924	Brazil	de Oliveira et al. 2007; Meneses et al. 2016; Oliveira et al. 2013a; Oliveira et al. 2013b; Waquil et al. 1999
D. maidis	-12.17932	-45.00745	Brazil	de Oliveira et al. 2007; Meneses et al. 2016; Oliveira et al. 2013a; Oliveira et al. 2013b; Waquil et al. 1999
D. maidis	-21.76810	-47.11003	Brazil	de Oliveira et al. 2007; Meneses et al. 2016; Oliveira et al. 2013a; Oliveira et al. 2013b; Waquil et al. 1999
D. maidis	-24.66452	-50.85886	Brazil	de Oliveira et al. 2007; Meneses et al. 2016; Oliveira et al. 2013a; Oliveira et al. 2013b; Waquil et al. 1999
D. maidis	-18.40543	-46.43889	Brazil	de Oliveira et al. 2007; Meneses et al. 2016; Oliveira et al. 2013a; Oliveira et al. 2013b; Waquil et al. 1999
D. maidis	-17.92380	-50.84200	Brazil	Santana Jr et al. 2019
D. maidis	-17.84450	-50.87100	Brazil	Santana Jr et al. 2019
D. maidis	-17.84370	-50.93080	Brazil	Santana Jr et al. 2019
D. maidis	-17.86030	-51.01700	Brazil	Santana Jr et al. 2019
D. maidis	-17.64210	-51.10620	Brazil	Santana Jr et al. 2019
D. maidis	-17.63670	-51.19470	Brazil	Santana Jr et al. 2019
D. maidis	-17.71120	-51.20510	Brazil	Santana Jr et al. 2019
D. maidis	-17.79310	-51.23140	Brazil	Santana Jr et al. 2019
D. maidis	-17.74170	-51.33470	Brazil	Santana Jr et al. 2019
D. maidis	-17.65790	-51.30010	Brazil	Santana Jr et al. 2019
D. maidis	-17.60650	-51.39610	Brazil	Santana Jr et al. 2019
D. maidis	-17.45810	-51.30050	Brazil	Santana Jr et al. 2019
D. maidis	-17.43310	-51.50360	Brazil	Santana Jr et al. 2019
D. maidis	-17.43330	-51.64580	Brazil	Santana Jr et al. 2019

D. maidis	-17.78280	-51.56820	Brazil	Santana Jr et al. 2019
D. maidis	-17.72750	-51.63690	Brazil	Santana Jr et al. 2019
D. maidis	-17.88870	-51.82820	Brazil	Santana Jr et al. 2019
D. maidis	-17.95230	-51.89770	Brazil	Santana Jr et al. 2019
D. maidis	-17.84550	-51.94840	Brazil	Santana Jr et al. 2019
D. maidis	-17.83460	-52.03110	Brazil	Santana Jr et al. 2019
D. maidis	-17.87330	-52.06260	Brazil	Santana Jr et al. 2019
D. maidis	-17.94970	-52.15570	Brazil	Santana Jr et al. 2019
D. maidis	-17.86310	-52.16620	Brazil	Santana Jr et al. 2019
D. maidis	-17.74790	-52.14970	Brazil	Santana Jr et al. 2019
D. maidis	-17.70420	-52.21960	Brazil	Santana Jr et al. 2019
D. maidis	3.51889	-76.31594	Colombia	Heady and Nault 1985; Triplehorn and Nault 1985
D. maidis	4.69154	-74.20591	Colombia	Heady and Nault 1985; Triplehorn and Nault 1985
D. maidis	4.71720	-74.24725	Colombia	Heady and Nault 1985; Triplehorn and Nault 1985
D. maidis	6.13049	-75.35549	Colombia	Heady and Nault 1985; Triplehorn and Nault 1985
D. maidis	6.30842	-75.59115	Colombia	Heady and Nault 1985; Triplehorn and Nault 1985
D. maidis	6.53875	-75.83035	Colombia	Heady and Nault 1985; Triplehorn and Nault 1985
D. maidis	6.95719	-75.42146	Colombia	Heady and Nault 1985; Triplehorn and Nault 1985
D. maidis	9.96615	-84.21354	Costa Rica	Heady and Nault 1985
D. maidis	10.00620	-84.26441	Costa Rica	Heady and Nault 1985
D. maidis	10.19181	-84.38720	Costa Rica	Heady and Nault 1985
D. maidis	10.49694	-84.52276	Costa Rica	Heady and Nault 1985
D. maidis	21.71020	-82.76160	Cuba	Genaro and Portuondo 1997
D. maidis	13.45011	-89.12415	El Salvador	Quezada 1979
D. maidis	14.17449	-91.31297	Guatemala	Zahniser 2007
D. maidis	14.30028	-91.57344	Guatemala	Zahniser 2007
D. maidis	14.33032	-89.69997	Guatemala	Zahniser 2007
D. maidis	14.33736	-91.03621	Guatemala	Zahniser 2007
D. maidis	14.06120	-86.29678	Honduras	Zahniser 2007
D. maidis	14.06345	-86.86405	Honduras	Zahniser 2007
D. maidis	14.13533	-86.24937	Honduras	Zahniser 2007
D. maidis	14.27982	-87.40029	Honduras	Zahniser 2007

D. maidis	17.99087	-76.89595	Jamaica	Eden-Green and Waters 1981
D. maidis	17.00201	-100.07512	Mexico	Bellota et al. 2018; Hidalgo et al. 1998; Moya-Raygoza 2012
D. maidis	17.02384	-97.93935	Mexico	Bellota et al. 2018; Hidalgo et al. 1998; Moya-Raygoza 2012
D. maidis	17.97825	-97.86692	Mexico	Bellota et al. 2018; Hidalgo et al. 1998; Moya-Raygoza 2012
D. maidis	18.36241	-99.87896	Mexico	Bellota et al. 2018; Hidalgo et al. 1998; Moya-Raygoza 2012
D. maidis	18.64850	-97.38848	Mexico	Bellota et al. 2018; Hidalgo et al. 1998; Moya-Raygoza 2012
D. maidis	18.81701	-97.20934	Mexico	Bellota et al. 2018; Hidalgo et al. 1998; Moya-Raygoza 2012
D. maidis	18.83663	-97.07482	Mexico	Bellota et al. 2018; Hidalgo et al. 1998; Moya-Raygoza 2012
D. maidis	18.83969	-96.41044	Mexico	Bellota et al. 2018; Hidalgo et al. 1998; Moya-Raygoza 2012
D. maidis	19.01535	-98.27882	Mexico	Bellota et al. 2018; Hidalgo et al. 1998; Moya-Raygoza 2012
D. maidis	19.12471	-96.14997	Mexico	Bellota et al. 2018; Hidalgo et al. 1998; Moya-Raygoza 2012
D. maidis	19.53172	-98.84592	Mexico	Bellota et al. 2018; Hidalgo et al. 1998; Moya-Raygoza 2012
D. maidis	19.79331	-104.21652	Mexico	Bellota et al. 2018; Hidalgo et al. 1998; Moya-Raygoza 2012
D. maidis	19.87824	-103.57315	Mexico	Bellota et al. 2018; Hidalgo et al. 1998; Moya-Raygoza 2012
D. maidis	12.33750	-86.68568	Nicaragua	Power 1989
D. maidis	-14.83840	-74.95657	Peru	Nault et al. 1981
D. maidis	-14.08513	-75.67685	Peru	Nault et al. 1981
D. maidis	-13.50392	-71.98201	Peru	Nault et al. 1981
D. maidis	-13.42854	-76.15044	Peru	Nault et al. 1981
D. maidis	-13.32306	-72.12728	Peru	Nault et al. 1981
D. maidis	-13.31969	-72.08228	Peru	Nault et al. 1981
D. maidis	-13.26032	-72.26837	Peru	Nault et al. 1981
D. maidis	-13.16857	-74.18673	Peru	Nault et al. 1981
D. maidis	-13.11426	-74.19028	Peru	Nault et al. 1981
D. maidis	-11.99103	-76.92950	Peru	Nault et al. 1981
D. maidis	-7.33324	-78.17762	Peru	Nault et al. 1981
D. maidis	-7.31868	-78.17204	Peru	Nault et al. 1981
D. maidis	-7.24388	-78.38309	Peru	Nault et al. 1981
D. maidis	-7.19995	-78.32386	Peru	Nault et al. 1981
D. maidis	-7.15629	-78.49803	Peru	Nault et al. 1981
D. maidis	-7.07980	-76.51103	Peru	Nault et al. 1981
D. maidis	17.97287	-66.26129	Puerto Rico	Zahniser 2007

D. maidis	26.14252	-98.22472	United States	Summers et al. 2004
D. maidis	26.60454	-80.58271	United States	Summers et al. 2004
D. maidis	31.31276	-92.51217	United States	Summers et al. 2004
D. maidis	31.45392	-83.46290	United States	Summers et al. 2004
D. maidis	33.66018	-93.66747	United States	Summers et al. 2004
D. maidis	33.86385	-91.44481	United States	Summers et al. 2004
D. maidis	35.25073	-92.69142	United States	Summers et al. 2004
D. maidis	36.11005	-119.89433	United States	Summers et al. 2004
D. maidis	36.11837	-118.86043	United States	Summers et al. 2004
D. maidis	36.75994	-119.51644	United States	Summers et al. 2004
D. maidis	8.79502	-69.75772	Venezuela	Clavijo and Notz 1978
D. maidis	9.91937	-67.38934	Venezuela	Clavijo and Notz 1978
D. maidis	4.61979	-76.01779	Colombia	Arcila Bohórquez et al. 2024
D. maidis	4.61464	-76.00470	Colombia	Arcila Bohórquez et al. 2024
D. maidis	4.62045	-75.99589	Colombia	Arcila Bohórquez et al. 2024
D. maidis	4.58304	-76.05802	Colombia	Arcila Bohórquez et al. 2024
D. maidis	4.62244	-76.02020	Colombia	Arcila Bohórquez et al. 2024
D. maidis	4.62244	-76.02020	Colombia	Arcila Bohórquez et al. 2024
D. maidis	4.62244	-76.02020	Colombia	Arcila Bohórquez et al. 2024
D. maidis	3.69702	-76.34142	Colombia	Arcila Bohórquez et al. 2024
D. maidis	3.66410	-73.57810	Colombia	Arcila Bohórquez et al. 2024
D. maidis	8.93044	-75.78902	Colombia	Arcila Bohórquez et al. 2024
D. maidis	3.99985	-73.38125	Colombia	Arcila Bohórquez et al. 2024
D. maidis	4.18639	-74.96396	Colombia	Arcila Bohórquez et al. 2024
D. maidis	4.19809	-74.90603	Colombia	Arcila Bohórquez et al. 2024
D. maidis	3.66437	-73.57928	Colombia	Arcila Bohórquez et al. 2024
D. maidis	4.21408	-72.48489	Colombia	Arcila Bohórquez et al. 2024
D. maidis	8.92274	-75.83205	Colombia	Arcila Bohórquez et al. 2024
D. maidis	3.51227	-76.28361	Colombia	Arcila Bohórquez et al. 2024
D. maidis	4.08928	-74.92521	Colombia	Arcila Bohórquez et al. 2024
D. maidis	3.50769	-73.72275	Colombia	Arcila Bohórquez et al. 2024
D. maidis	3.51141	-76.33840	Colombia	Arcila Bohórquez et al. 2024

D. maidis	4.37619	-76.14038	Colombia	Arcila Bohórquez et al. 2024
D. maidis	8.93050	-75.78886	Colombia	Arcila Bohórquez et al. 2024
D. maidis	4.17862	-74.89360	Colombia	Arcila Bohórquez et al. 2024
D. maidis	4.45448	-71.52541	Colombia	Arcila Bohórquez et al. 2024
D. maidis	7.89149	-73.50061	Colombia	Arcila Bohórquez et al. 2024
D. maidis	10.63308	-73.12482	Colombia	Arcila Bohórquez et al. 2024
D. maidis	2.668382	-75.333302	Colombia	Reinoso, 2021
D. maidis	2.869621	-75.281099	Colombia	Reinoso, 2021
D. maidis	2.225862	-75.611987	Colombia	Reinoso, 2021
D. maidis	2.108519	-75.831831	Colombia	Reinoso, 2021
D. maidis	12.14722	-86.16278	Nicaragua	GBIF, 2025
D. maidis	43.08991	-89.37417	United States	GBIF, 2025
D. maidis	43.08991	-89.37417	United States	GBIF, 2025
D. maidis	43.30743	-89.91241	United States	GBIF, 2025
D. maidis	39.52448	-79.81770	United States	GBIF, 2025
D. maidis	39.23004	-86.42012	United States	GBIF, 2025
D. maidis	39.52443	-79.81761	United States	GBIF, 2025
D. maidis	42.61599	-83.19203	United States	GBIF, 2025
D. maidis	42.20844	-93.42352	United States	GBIF, 2025
D. maidis	41.21864	-75.84174	United States	GBIF, 2025
D. maidis	39.52441	-79.81753	United States	GBIF, 2025
D. maidis	39.52448	-79.81770	United States	GBIF, 2025
D. maidis	42.58745	-83.19291	United States	GBIF, 2025
D. maidis	39.52443	-79.81757	United States	GBIF, 2025
D. maidis	35.02559	-85.36419	United States	GBIF, 2025
D. maidis	35.14585	-79.01185	United States	GBIF, 2025
D. maidis	-7.32944	-71.84611	Brazil	GBIF, 2025
D. maidis	-31.38078	-64.20966	Argentina	GBIF, 2025
D. maidis	30.35750	-97.72340	United States	GBIF, 2025
D. maidis	32.24645	-110.88481	United States	GBIF, 2025
D. maidis	-27.57670	-56.67837	Argentina	GBIF, 2025
D. maidis	37.50326	-119.96137	United States	GBIF, 2025

D. maidis	28.26229	-105.49278	México	GBIF, 2025
D. maidis	16.81667	-92.96667	México	GBIF, 2025
D. maidis	33.26590	-88.87070	United States	GBIF, 2025
D. maidis	33.26590	-88.87070	United States	GBIF, 2025
D. maidis	16.81667	-92.96667	México	GBIF, 2025
D. maidis	33.26590	-88.87070	United States	GBIF, 2025
D. maidis	33.26590	-88.87070	United States	GBIF, 2025
D. maidis	33.26590	-88.87070	United States	GBIF, 2025
D. maidis	33.26590	-88.87070	United States	GBIF, 2025
D. maidis	19.66833	-104.03139	México	GBIF, 2025
D. maidis	19.05000	-104.26667	México	GBIF, 2025
D. maidis	19.98333	-97.18333	México	GBIF, 2025
D. maidis	19.66833	-104.03139	México	GBIF, 2025
D. maidis	18.20130	-67.14510	Puerto Rico	GBIF, 2025
D. maidis	18.20130	-67.14510	Puerto Rico	GBIF, 2025
D. maidis	18.20130	-67.14510	Puerto Rico	GBIF, 2025
D. maidis	18.20130	-67.14510	Puerto Rico	GBIF, 2025
D. maidis	4.29860	-74.80460	Colombia	GBIF, 2025
D. maidis	18.20130	-67.14510	Puerto Rico	GBIF, 2025
D. maidis	19.48700	-70.61500	Dominican Republic	GBIF, 2025
D. maidis	18.20130	-67.14510	Puerto Rico	GBIF, 2025
D. maidis	18.20130	-67.14510	Puerto Rico	GBIF, 2025
D. maidis	19.48700	-70.61500	Dominican Republic	GBIF, 2025
D. maidis	4.29860	-74.80460	Colombia	GBIF, 2025
D. maidis	18.20130	-67.14510	Puerto Rico	GBIF, 2025
D. maidis	19.48700	-70.61500	Dominican Republic	GBIF, 2025
D. maidis	19.48700	-70.61500	Dominican Republic	GBIF, 2025
D. maidis	4.34500	-74.36100	Colombia	GBIF, 2025
D. maidis	19.48700	-70.61500	Dominican Republic	GBIF, 2025
D. maidis	19.48700	-70.61500	Dominican Republic	GBIF, 2025
D. maidis	18.20130	-67.14510	Puerto Rico	GBIF, 2025
D. maidis	19.48700	-70.61500	Dominican Republic	GBIF, 2025

D. maidis	15.43200	-87.90400	Honduras	GBIF, 2025
D. maidis	18.20130	-67.14510	Puerto Rico	GBIF, 2025
D. maidis	19.48700	-70.61500	Dominican Republic	GBIF, 2025
D. maidis	18.20130	-67.14510	Puerto Rico	GBIF, 2025
D. maidis	19.48700	-70.61500	Dominican Republic	GBIF, 2025
D. maidis	19.48700	-70.61500	Dominican Republic	GBIF, 2025
D. maidis	18.20130	-67.14510	Puerto Rico	GBIF, 2025
D. maidis	19.48700	-70.61500	Dominican Republic	GBIF, 2025
D. maidis	19.48700	-70.61500	Dominican Republic	GBIF, 2025
D. maidis	30.31070	-91.09616	United States	GBIF, 2025
D. maidis	40.35880	-91.41880	United States	GBIF, 2025
D. maidis	31.63464	-98.24814	United States	GBIF, 2025
D. maidis	31.63464	-98.24814	United States	GBIF, 2025
D. maidis	30.35750	-97.72340	United States	GBIF, 2025
D. maidis	41.75047	-91.53995	United States	GBIF, 2025
D. maidis	32.31177	-97.01598	United States	GBIF, 2025
D. maidis	33.03536	-96.72633	United States	GBIF, 2025
D. maidis	30.36642	-97.67348	United States	GBIF, 2025
D. maidis	31.63464	-98.24814	United States	GBIF, 2025
D. maidis	31.63464	-98.24814	United States	GBIF, 2025
D. maidis	18.60095	-98.84515	México	GBIF, 2025
D. maidis	18.60095	-98.84515	México	GBIF, 2025
D. maidis	40.00700	-83.03000	United States	GBIF, 2025
D. maidis	45.3999	-72.8518	Canada	Jacques et al. 2025 (in preparation)
D. maidis	45.5868	-73.0612	Canada	Jacques et al. 2025 (in preparation)
D. maidis	46.3976	-72.7646	Canada	Jacques et al. 2025 (in preparation)
D. maidis	46.4552	-71.7505	Canada	Jacques et al. 2025 (in preparation)
D. maidis	45.5117	-72.9778	Canada	Jacques et al. 2025 (in preparation)
D. maidis	42.2092	-93.4244	United States	Current study
D. maidis	36.13339	-97.10624	United States	Current study
D. maidis	35.03601	-97.90017	United States	Current study
D. maidis	37.01117	-93.59683	United States	Current study

D. maidis	38.90684	-92.28218	United States	Current study
D. elimatus	18.7149	-97.3408	México	GBIF, 2025
D. elimatus	19.8333	-99.9833	México	GBIF, 2025
D. elimatus	19.3000	-98.7333	México	GBIF, 2025
D. elimatus	19.8333	-99.9833	México	GBIF, 2025
D. elimatus	21.5333	-101.7167	México	GBIF, 2025
D. elimatus	20.0500	-98.5333	México	GBIF, 2025
D. elimatus	19.3000	-98.7333	México	GBIF, 2025
D. elimatus	19.8333	-99.9833	México	GBIF, 2025
D. elimatus	20.1667	-98.3000	México	GBIF, 2025
D. elimatus	19.8333	-99.9833	México	GBIF, 2025
D. elimatus	20.1667	-98.3000	México	GBIF, 2025
D. elimatus	19.8333	-99.9833	México	GBIF, 2025
D. elimatus	19.3000	-98.7333	México	GBIF, 2025
D. elimatus	19.8081	-101.6965	México	GBIF, 2025
D. elimatus	19.8079	-101.6961	México	GBIF, 2025
D. elimatus	18.6009	-98.8451	México	GBIF, 2025
D. elimatus	18.6009	-98.8451	México	GBIF, 2025
D. elimatus	19.7943	-104.2225	México	Moya-Raygoza et al 2014
D. elimatus	21.2336	-103.5003	México	Moya-Raygoza et al 2014
D. elimatus	19.6073	-98.9254	México	Nault and Madden, 1985
D. elimatus	21.4751	-103.0929	México	Moya-Raygoza, 2002
D. elimatus	16.3635	-96.6379	México	Moya-Raygoza, 2002
D. elimatus	17.8517	-96.2151	México	Moya-Raygoza, 2002
D. elimatus	18.4848	-97.4594	México	Moya-Raygoza, 2002
D. elimatus	17.2465	-96.8607	México	Moya-Raygoza, 2002
D. elimatus	17.4531	-97.2368	México	Moya-Raygoza, 2002
D. elimatus	19.6761	-103.9343	México	Moya-Raygoza, 2002
D. elimatus	20.3270	-102.0474	México	Moya-Raygoza, 2002
D. elimatus	20.6488	-101.3146	México	Moya-Raygoza, 2002
D. elimatus	20.5567	-100.6898	México	Moya-Raygoza, 2002
D. elimatus	20.2319	-98.9158	México	Moya-Raygoza, 2002

D. elimatus	19.8779	-100.4407	México	Moya-Raygoza, 2002
D. elimatus	20.2207	-99.2084	México	Moya-Raygoza, 2002
D. elimatus	19.8719	-103.5955	México	Moya-Raygoza, 2007
D. elimatus	31.3629	-109.6696	United States	Triplehorn and Nault, 1985
D. elimatus	31.4682	-108.5139	United States	Triplehorn and Nault, 1985
D. elimatus	19.4923	-98.8775	Mexico	Maramorosch, 1959
D. elimatus	19.6218	-98.9146	Mexico	Heady and Nault, 1985
MBS	-23.1669	-48.0503	Brazil	Orlovskis et al. 2017
MBS	-20.5336	-48.51694	Brazil	Orlovskis et al. 2017
MBS	-19.7669	-44.40028	Brazil	Orlovskis et al. 2017
MBS	18.6797	-99.1164	Mexico	Moya-Raygoza and Nault, 1998
MBS	19.5287	-98.8484	Mexico	Moya-Raygoza and Nault, 1998
MBS	20.4901	-97.5463	Mexico	Moya-Raygoza and Nault, 1998
MBS	19.7329	-97.5754	Mexico	Perez-Lopez et al. 2016
MBS	19.7586	-97.5509	Mexico	Perez-Lopez et al. 2016
MBS	19.7323	-97.5970	Mexico	Perez-Lopez et al. 2016
MBS	19.8930	-97.5874	Mexico	Perez-Lopez et al. 2016
MBS	19.6455	-97.1047	Mexico	Perez-Lopez et al. 2016
MBS	-12.0174	-75.2889	Peru	Gamarra et al. 2022
MBS	-11.9937	-75.2340	Peru	Gamarra et al. 2022
MBS	-11.9712	-75.2614	Peru	Gamarra et al. 2022
MBS	-11.9562	-75.2911	Peru	Gamarra et al. 2022
MBS	-12.1206	-75.2537	Peru	Gamarra et al. 2022
MBS	-12.0982	-75.2905	Peru	Gamarra et al. 2022
MBS	-12.079	-75.3254	Peru	Gamarra et al. 2022
MBS	-12.0493	-75.2902	Peru	Gamarra et al. 2022
MBS	-11.8344	-75.4050	Peru	Gamarra et al. 2022
MBS	-10.4923	-48.3144	Brazil	Toloy et al. 2024
MBS	-12.2160	-46.0381	Brazil	Galvao et al. 2020
MBS	-17.8504	-50.9295	Brazil	Galvao et al. 2020
MBS	-18.0182	-49.3846	Brazil	Galvao et al. 2020
MBS	-17.6275	-47.7885	Brazil	Galvao et al. 2020

MBS	-15.8446	-43.3385	Brazil	Galvao et al. 2020
MBS	-18.9711	-46.9784	Brazil	Galvao et al. 2020
MBS	-21.4844	-47.0473	Brazil	Galvao et al. 2020
MBS	-21.7788	-47.1220	Brazil	Galvao et al. 2020
MBS	-23.4427	-48.9035	Brazil	Galvao et al. 2020
MBS	-28.6303	-53.6330	Brazil	Stürmer et al. 2024
MBS	-20.0322	-45.9868	Brazil	Da Cunha et al. 2023
MBS	-26.1874	-50.4264	Brazil	Canale et al. 2024
MBS	-22.6906	-50.4770	Brazil	Gomes et al. 2004
MBS	-24.0225	-48.3687	Brazil	Gomes et al. 2004
MBS	-22.3101	-54.8318	Brazil	Gomes et al. 2004
MBS	-21.0237	-47.8078	Brazil	Gomes et al. 2004
MBS	26.4341	-98.2210	United States	Harrison et al. 1996
MBS	25.4227	-80.5491	United States	Harrison et al. 1996
MBS	10.6634	-85.5146	Costa Rica	Harrison et al. 1996
MBS	20.5805	-97.4575	Mexico	Harrison et al. 1996
MBS	18.6831	-99.1284	Mexico	Harrison et al. 1996
MBS	4.692313	-75.963432	Colombia	Dr. Javier Orozco, Alphanalitica S.A.S, 2022
MBS	3.855608	-76.308398	Colombia	Dr. Javier Orozco, Alphanalitica S.A.S, 2022
MBS	3.348848	-76.228093	Colombia	Dr. Javier Orozco, Alphanalitica S.A.S, 2022
MBS	2.668382	-75.333302	Colombia	Reinoso, 2021
MBS	2.869621	-75.281099	Colombia	Reinoso, 2021
MBS	2.225862	-75.611987	Colombia	Reinoso, 2021
MBS	2.108519	-75.831831	Colombia	Reinoso, 2021
MBS	42.2092	-93.4244	United States	Current study
MBS	36.13339	-97.10624	United States	Current study
MBS	35.03601	-97.90017	United States	Current study

References

Arcila Bohórquez C, Rodriguez-Chalarca J, Bernal A, Reyes Caldas PA, and Molina Escobar JA (2024). Epidemiología del complejo del achaparramiento del maíz en las zonas agroecológicas de Colombia. Available from: https://alliancebioversityciat.org/publications-data/epidemiologia-del-complejo-del-achaparramiento-del-maiz-en-las-zonas

Bellota E, Dávila-Flores A, Bernal JS (2018). A bird in the hand versus two in the bush? The specialist leafhopper *Dalbulus maidis* (Hemiptera: Cicadellidae) does not discriminate against sub-optimal host plants (*Zea* spp.). Neotropical Entomology 47:171-180 doi:10.1007/s13744-017-0516-0

Canale MC et al. (2024). *Leptodelphax maculigera* (Hemiptera: Delphacidae) harbors the corn stunt complex pathogens. Plant Disease 108(9):2653-2657.

Clavijo S, Notz A (1978). Fluctuaciones poblacionales en maiz de *Spodoptera frugiperda*, *Delphax maidis* y *Oalbulus maidis*, en San Nicolas, estado Portuguesa, Venezuela, bajo condiciones de epoca lluviosa. Boletín de Entomología Venezolana 1:1-20 doi:http://entomotropica.org/index.php/bev/article/view/612

Da Cunha et al. (2023). Distribution of *Dalbulus maidis* (DeLong) (Hemiptera: Cicadellidae) and incidence of maize rayado fino virus and *Candidatus* Phytoplasma asteris in corn succession planting systems. Pest Management Science 79(7):2325-2337.

de Oliveira CM, Lopes JRS, Camargo LEA, Fungaro MHP, Nault LR (2007). Genetic diversity in populations of *Dalbulus maidis* (DeLong and Wolcott) (Hemiptera: Cicadellidae) from distant localities in Brazil assessed by Rapd-Pcr markers. Environmental Entomology 36:204-212 doi:10.1603/0046-225X(2007)36[204:GDIPOD]2.0.CO;2

Eden-Green BSJ, Waters H (1981). Isolation of corn stunt spiroplasma in Jamaica, and probable dual infection with maize mosaic virus. Annals of Applied Biology 99:129-134 doi:10.1111/j.1744-7348.1981.tb05139.x

Galvão SR, Sabato EO, Bedendo IP (2020). Occurrence and distribution of single or mixed infection of phytoplasma and spiroplasma causing corn stunting in Brazil. Tropical Plant Pathology 46:152-155

Gamarra DG et al. (2022). Diverse phytoplasmas associated with maize bushy stunt disease in Peru. European Journal of Plant Pathology 163:223-235.

Genaro JA, Portuondo E (1997). An annotated preliminary checklist of the Dryinidae of Cuba (Insecta: Hymenoptera). Caribbean Journal of Science, 33 (1–2):112–114.

Global Biodiversity Information Facility (GBIF). *Dalbulus maidis* occurrence records. GBIF.org (7 July 2025) GBIF Occurrence Download https://doi.org/10.15468/dl.9hgne2

Global Biodiversity Information Facility (GBIF). *Dalbulus elimatus* occurrence records. GBIF.org (7 July 2025) GBIF Occurrence Download https://doi.org/10.15468/dl.dg2jpg

Gomes EA et al. (2004). Genetic variability of Brazilian phytoplasma and spiroplasma isolated from maize plants. Pesquisa Agropecuaria Brasileira 39(1):61-65.

Harrison NA et al. (1996). PCR assay for detection of the phytoplasma associated with maize bushy stunt disease. Plant Disease 80(3):263-269.

Heady SE, Nault LR (1985). Escape behavior of *Dalbulus* and *Baldulus* leafhoppers (Homoptera: Cicadellidae). Environmental Entomology 14:154-158 doi:10.1093/ee/14.2.154

Hidalgo H, jeffers D, Castanon G, Rodriguez F (1998). Resistencia al achaparramiento del maíz mediante infestaciones de *Dalbulus maidis* en maíz. Agronomía Mesoamericana 9(2): 119-124.

Maramorosch K (1959). An ephemeral disease of maize transmitted by *Dalbulus elimatus*. Entomologia Experimentalis et Applicata 2 (2): 169-170.

Meneses AR, Querino RB, Oliveira CM, Maia AHN, Silva PRR (2016). Seasonal and vertical distribution of *Dalbulus maidis* (Hemiptera: Cicadellidae) in brazilian corn fields. Florida Entomologist 99:750-754 doi:10.1653/024.099.0428

Moya-Raygoza G, Nault LR (1998). Transmission biology of maize bushy stunt phytplasma by the corn leafhopper (Homoptera: Cicadellidae). Annals of the Entomological Society of America 91(5):668-676.

Moya-Raygoza G (2002). Distribución y hábitats de *Dalbulus* spp. (Homoptera: Cicadellidae) durante la estación seca en México. Acta Zoologica Mexicana 85:119-128.

Moya-Raygoza G (2007). Native parasitoids of *Dalbulus elimatus* and *Dalbulus maidis* (Hemiptera: Cicadellidae) in winter crops of central Mexico. The Canandian Entomologist 139:722-727.

Moya-Raygoza G (2012). Native parasitoids of *Dalbulus elimatus* and *Dalbulus maidis* (Hemiptera: Cicadellidae) in winter crops of central Mexico. The Canadian Entomologist 139:722-727 doi:10.4039/n06-053

Moya-Raygoza G, Renteria I, Albarracin ER, Virla EG (2014). Egg parasitoids of the leafhoppers *Dalbulus maidis* and *Dalbulus elimatus* (Hemiptera: Cicadellidae) in two maize habitats. Florida Entomologist 97(1):309-312.

Nault LR, Gordon DT, Loayza JC (1981). Maize virus and mycoplasma diseases in Peru. Tropical Pest Management 27:363-369 doi:10.1080/09670878109413806

Nault LR, Madden LV (1985). Ecological strategies of *Dalbulus* leafhoppers. Ecological Entomology 10:57-63.

Oliveira CM, Lopes JRS, Nault LR (2013a). Survival strategies of *Dalbulus maidis* during maize off-season in Brazil. Entomologia Experimentalis et Applicata 147:141-153 doi:10.1111/eea.12059

Oliveira CMD et al. (2013b). Abundance and species richness of leafhoppers and planthoppers (Hemiptera: Cicadellidae and Delphacidae) in brazilian maize crops. Florida Entomologist 96:1470-1481 doi:10.1653/024.096.0427

Orlovskis Z et al. (2017). A few sequence polymorphisms among isolates of Maize bushy stunt phytoplasma associate with organ proliferation symptons of infected maize plants. Annals of Botany 119:869-884.

Pérez-López E et al. (2016). Maize bushy stunt phytoplasma affects native corn at high elevations in Southeast Mexico. European Journal of Plant Pathology 145:963-971

Power AG (1989). Influence of plant spacing and nitrogen fertilization in maize on *Dalbulus maidis* (Homoptera: Cicadellidae), vector of corn stunt. Environmental Entomology 18:494-498 doi:10.1093/ee/18.3.494

Quezada JR Hallazgo DE (1979). *Agonatopus* sp: (Hymenoptera: Dryinidae), parasito del *Dalbulus maidis* (Homoptera: Cicadellidae) en El Salvador. In: VI Reunión Nacional de Control Biológico, Culiacán, México.

Reinoso IS (2021). Caracterización poblacional de Dalbulus sp. (Hemiptera: Cicadellidae) insecto vector de Mollicutes en maíz, en el departamento del Huila. Universidad Nacional de Colombia – Master thesis. 110p.

Santana Jr PA et al. (2019). Assessing the impact of climate change on the worldwide distribution of *Dalbulus maidis* (DeLong) using MaxEnt. Pest Management Science 75(10):2706-2715 doi10.1002/ps.5379

Stumer GR et al. (2024). Simultaneous infection of *Leptodelphax maculigera* (Hemiptera: Delphacidae) by maize rayado fino virus (MRFV) and maize bushy stunt phytoplasma (MPSP) detected in Rio Grande do Sul, Brazil. Journal of Plant Diseases and Protection 131:2247-2253

Summers CG, Newton JAS, Opgenorth DC (2004). Overwintering of corn leafhopper, *Dalbulus maidis* (Homoptera: Cicadellidae), and Spiroplasma kunkelii (Mycoplasmatales: Spiroplasmataceae) in California's San Joaquin Valley. Environmental Entomology 33:1644-1651 doi:10.1603/0046-225X-33.6.1644

Triplehorn BW, Nault LR (1985). Phylogenetic classification of the genus *Dalbulus* (Homoptera: Cicadellidae), and notes on the phylogeny of the Macrostelini Annals of the Entomological Society of America 78:291-315 doi:10.1093/aesa/78.3.291

Toloy RS et al. (2024). Exploring natural infection in alternative hosts of corn stunt spiroplasma and tracking the temporal dynamics of corn stunt disease in the Brazilian Cerrado Biome. Agronomy 14:2341.

Virla EG, Casuso M, Frias EA (2010). A preliminary study on the effects of a transgenic corn event on the non-target pest *Dalbulus maidis* (Hemiptera: Cicadellidae) Crop Protection 29:635-638 doi:https://doi.org/10.1016/j.cropro.2009.12.019

Virla EG, Moya-Raygoza G, Luft-Albarracin E (2013). Egg parasitoids of the corn leafhopper, *Dalbulus maidis*, in the southernmost area of its distribution range Journal of Insect Science 13:10-10 doi:10.1673/031.013.1001

Waquil JM, Viana PA, Cruz I, Santos JP (1999). Aspectos da biologia da cigarrinha-do-milho, *Dalbulus maidis* (DeLong & Wolcott) (Hemiptera: Cicadellidae) Anais da Sociedade Entomológica do Brasil 28:413-420

Zahniser J (2007) An online interactive key and searchable database of Deltocephalinae (Hemiptera: Cicadellidae). http://zahniser.speciesfile.org/.

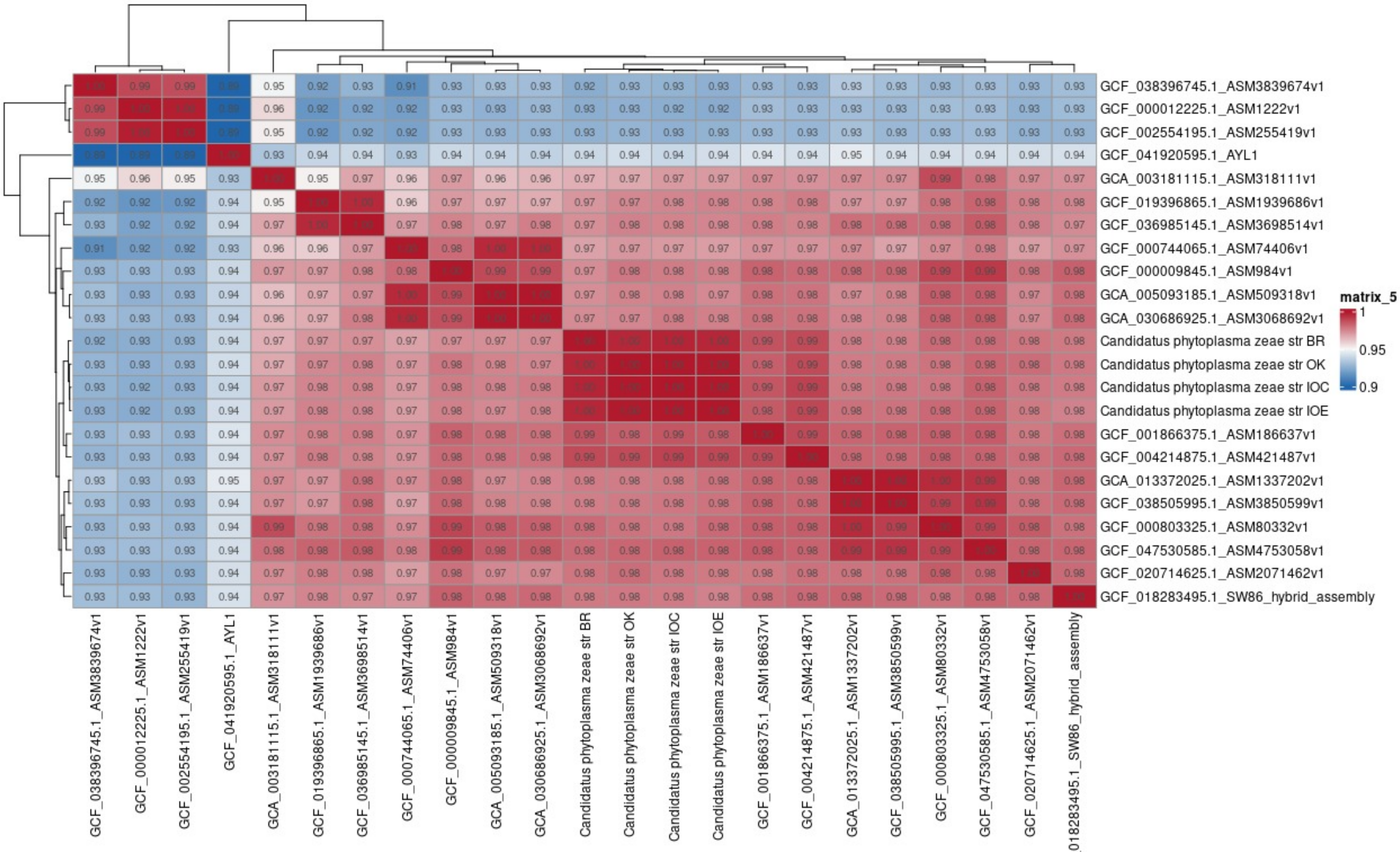
GCF_038396745.1_ASM3839674v1
GCF_000012225.1_ASM1222v1
GCF_002554195.1_ASM255419v1
GCF_041920595.1_AYL1
GCA_003181115.1_ASM318111v1
GCF_019396865.1_ASM1939686v1
GCF_036985145.1_ASM3698514v1
GCF_000744065.1_ASM74406v1
GCF_000009845.1_ASM984v1
GCA_005093185.1_ASM509318v1
GCA_030686925.1_ASM3068692v1
Candidatus phytoplasma zeae str BR
Candidatus phytoplasma zeae str OK
Candidatus phytoplasma zeae str IOC
Candidatus phytoplasma zeae str IOE
GCF_001866375.1_ASM186637v1
GCF_004214875.1_ASM421487v1
GCA_013372025.1_ASM1337202v1
GCF_038505995.1_ASM3850599v1
GCF_000803325.1_ASM80332v1
GCF_047530585.1_ASM4753058v1
GCF_020714625.1_ASM2071462v1
GCF_018283495.1_SW86_hybrid_assembly
matrix_5
1
0.95
0.9

Figure S1. Genome-wide average nucleotide identity matrix between the three '*Ca*. P. zeae' genomes generated in this study and all available '*Ca*. P. asteris'-related strains genomes available in NCBI. In all cases the values are rounded up, final values are presented in **Table S6**.

The four '*Ca*. P. asteris genomes presenting **<95% genome-wide ANI identity** relative to '*Ca*. P. zeae' have been clearly validated as 16SrI and '*Ca*. P. asteris' -related strains. Additionally, the quality of the sequences is optimal with several of them assembled in the last 5 years, as the references below can confirm:

References:

- **GCA_038396745.1:** Toth, R., Ilic, A.-M., Huettel, B., Duduk, B., & Kube, M. 2024. Divergence within the taxon '*Candidatus* Phytoplasma asteris' confirmed by comparative genome analysis of carrot strains. Microorganisms, 12, 1016. https://doi.org/10.3390/microorganisms12051016
- **GCA_000012225.1:** Bai, X., Correa, V. R., Toruño, T. Y., Ammar, E.-D., Kamoun, S., & Hogenhout, S. A. 2009. AY-WB phytoplasma secretes a protein that targets plant cell nuclei. Molecular Plant-Microbe Interactions, 22(1), 18–30. https://doi.org/10.1094/MPMI-22-1-0018
- **GCA_002554195.1:** Sparks, M. E., Bottner-Parker, K. D., Gundersen-Rindal, D. E., & Lee, I.-M. 2018. Draft genome sequence of the New Jersey aster yellows strain of '*Candidatus* Phytoplasma asteris'. PLoS ONE, 13(2), e0192379. https://doi.org/10.1371/journal.pone.0192379
- **GCA_041920595.1:** Song, S., Wang, Q., Huo, L., Xie, L., Chen, J., Cui, H., Dai, Z., Kang, J., Li, Y., Guo, W., Chen, J., Kang, L., & Zhang, X. 2025. The phytoplasma ('*Candidatus* Phytoplasma arecae') is the crucial pathogen causing areca palm yellow leaf disease. Science Bulletin, 70(6), 847–851. https://doi.org/10.1016/j.scib.2024.10.020
